# Unsuspected adverse effect of albumin in severe ovarian hyperstimulation syndrome: a case report

**DOI:** 10.5935/1518-0557.20190002

**Published:** 2019

**Authors:** Natalia Darii, Milenko Pavlovic, Bogdan Doroftei, Anton Emil

**Affiliations:** 1 Department of Gynecology, CHU de Charleroi, Montigny-le-Tilleul, Belgium; 2 Department of Gynecology, Faculty of Medicine, Pontificia Universidad Católica de Chile, Santiago, Chile; 3 Center for Gynecologic Laparoscopic Surgery (CECLAG), Department of Gynecology, Hospital Clínico La Florida, Santiago, Chile; 4 Department of Gynecology, University of Medicine and Pharmacology Gr T Popa Iasi, Romania

**Keywords:** OHSS, ovarian hyperstimulation syndrome, management of OHSS, albumin

## Abstract

Ovarian hyperstimulation syndrome (OHSS) is one of the most serious complications of *in vitro* fertilization (IVF). We present a case of OHSS maintained and aggraved by albumin administration. A 29-year-old woman with severe OHSS was treated with albumin perfusion according to the guidelines. The albumin was administered in order to maintain intravascular oncotic pressure and to reverse the shift of fluid from the intravascular to the third space, but this therapeutic measure resulted in inadvertent maintenance of the syndrome. The treatment of OHSS is a delicate balance between invasive approaches, such as paracentesis, administration of colloids and minimal therapeutic intervention, particularly during pregnancy.

## INTRODUCTION

OHSS is one of the most severe complications of ovarian stimulation for assisted reproductive technology and must be classified as mild, moderate or severe. The prevalence of the severe form is 0.1-2.5% (Sansone *et al*., 2011), and IT remains unchanged despite progress in prevention and treatment approaches. Many hypotheses may explain the physiopathology of OHSS. VEGF produced by mature follicles or by luteinized granulosa cells appears to be involved in OHSS development (Abramov *et al*., 1997). VEGF promotes extra-vascular fluid shift by increasing vascular permeability, which could lead to hypovolemia, hemoconcentration, ascites, pleural and pericardial effusions and electrolytic imbalances (Sansone *et al*., 2011; RCOG, 2016). Several strategies have been described, depending on the OHSS severity. The most common treatment in severe cases is the intravenous administration of macromolecules like human albumin or hydroxyethyl starch. These hyperosmotic agents help maintain intravascular volume by increasing the intravascular oncotic pressure, thus drawing third-space fluid back into the intravascular space (Humaidan *et al*., 2010). We report the case of an OHSS-patient whom treatment with albumin could have been resulted in inadvertent maintenance of this syndrome.

## CASE REPORT

A 29-year-old woman (G0P0) underwent controlled ovarian stimulation using an antagonist protocol (Cetrotide™ 0.25mg, Merck Serono, London UK) after being informed that *in vitro* fertilization (IVF) was indicated due to male factor infertility (severe oligo-astheno-teratozoospermia). Her ovarian reserve showed an antral follicle count (AFC) of 17 and a follicle-stimulating hormone (FSH) level of 4.5mIU/mL. Recombinant FSH (Gonal-F™, Merck Serono, London, UK) was administered for a period of 13 days, with a total dose of 3,487.5IU. On day 6, ultrasound examination showed 10 ovarian follicles measuring more than 7mm in diameter and 14 ovarian follicles measuring less than 5mm in diameter for both ovaries, with an estradiol level of 198pg/ml. The patient did not report any abdominal discomfort or dyspnea.

The FSH antagonist cetrorelix (Cetrotide™ 0.25mg, Merck Serono, London, UK) was added on day 9 when at least one follicle measured 13mm in diameter. Ovulation was induced by the administration of 10,000IU hCG (Pregnyl™, Merck & Co., Brussels, Belgium) on day 13 when the estradiol level was 898pg/dl and 16 follicles measured more than 15mm in diameter. According to Kovács *et al*. (2006), when the estradiol level is within a comfortable level (between 1,500 and 2,500pg/ml), human chorionic gonadotropin can be administered and the cycle will continue.

Two days later, 24 oocytes were retrieved because small follicles were also aspirated. After fertilization, nine embryos were obtained. On the day of embryo transfer, no clinical or ultrasound signs of OHSS were detected. Despite the high number of oocytes retrieved, we decided to transfer only one 10-cell-stage embryo after counselling the patient.

Seven days after oocyte pick-up, the patient was admitted to the emergency department for abdominal pain, bloating, nausea and dyspnea, indicative of OHSS. Clinical examination revealed blood pressure of 90/60mmHg, a heart rate of 94/min, oxygen saturation of 96%, and a temperature of 36.6ºC. Ultrasound examination showed comparable ovarian size (75x48x51mm for the right ovary and 71x57x63mm for the left ovary), but significant ascites in the Douglas pouch (81x27x12mm), in the Retzius space (72x73x57mm) and around the liver (95x43mm). According to the SOGC-CFAS clinical practice guidelines (Shmorgun *et al*., 2011), the OHSS was classified as severe, based on hemoconcentration, hyponatremia, elevated liver enzymes, presence of significant ascites, pleural effusion and clinical symptoms.

**Figure 1:**
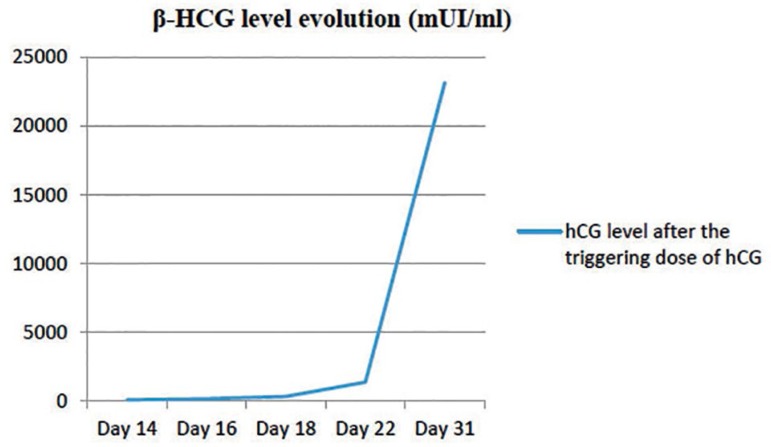
hCG evolution

The patient was admitted for intensive follow-up. We administered NaCl 0.9% perfusion in order to correct the electrolytic balance, as well as low-molecular-weight heparin nadroparin 0.3ml/day (Fraxiparine®, GSK, Inc) and compressive stockings for thromboembolic prevention. Due to severe dyspnea, thoracocentesis was carried out on day one (admission) and intravenous albumin was given. Pleural and abdominal ascites were drained. Pregnancy was confirmed by positive hCG (92.5mIU/ml). hCG evolution was correct (Fig. 1) and vaginal ultrasound performed a few days later revealed a single intrauterine gestational sac.

Seven paracenteses (between 1,000 and 2,100ml of exudate) and 5 thoracocenteses (between 1,100 and 1,600ml of exudate) under ultrasound guidance were required to decrease patient dyspnea and improve her comfort. A total of 19,900ml of exudate was extracted by the two procedures. Crystalloids (normal saline 1,000ml a day) and colloids (human albumin 20% 100ml twice a day then 100ml three times a day) were administered to counterbalance the loss of osmotic pressure related to albumin loss in the third space, but effusions persisted despite this strategy (Table 1). We finally suspected that the pleural and abdominal effusions were being sustained by our initial treatment. It was decided to stop albumin perfusion after 24 days of the admission. Surprisingly, the patient’s clinical and paraclinical status improved within 4 days of stopping albumin, with reduction of ascites and pleural fluid synthesis. She was subsequently discharged on day 29 (day 35 after HCG injection) without dyspnea and gave birth to a 2.800g girl at term and by the vaginal route.

**Table 1:** Patient evaluation

date	Hb	Hct	L	Alb level	HCG	P + or T	perf	albuminadm	T (ºC)	ovarian size (cm)	patient weight
Day7	16.1	45.5	8.06	3033		0			36.6	74x48x51(L); 71x57x63(R)	
Day8	13.5	38.1	7.74	2010		3000	3500	2	36.8		55.5
Day9	12.1	34.6	7.74	2495		2000	3500	2	36.5		54.4
Day10						0	1000	2	36.2		55.0
Day11	12.3	35.8	7.74	2997		1900	1000	2	37.3		55.0
Day12						0	1000	2	37.3		55.7
Day13						0	1000	2	36.9		55.4
Day14	13.8	40.9	7.74	2954	92.5	1400	1000	2	36.9		56.0
Day15						0	1000	2	36.0		55.5
Day16					158.3	1900	1000	2	36.9		55.6
Day17						0	1000	2	37.2		57.1
Day18	12.3	35.9	9.13	2949	333.3	1000	1000	2	37.1		57.1
Day19						0	1000	2	37.0		56.9
Day20						0	1000	2	37.0		55.9
Day21						0	1000	3	36.8		55.4
Day22	10.5	30.8	6.62		1381.2	1900	1000	3	37.2		55.5
Day23	10.9	32.1	6.55	3607		1500	1000	3	37.2		56.1
Day24						0	1000	3	37.3		56.9
Day25	10.2	30.1	6.08	3774		1100	1000	3	36.7		56.0
Day26						1100	1000	3	36.6		56.4
Day27						0	1000	3	37.3		57.9
Day28	10.1	29.6	6.61	4300		1400	1000	2	37.0	64x69x76(L); 120x76x74(R)	57.6
Day29						0	1000	2	36.8		56.9
Day30						0	1000	2	37.0		57.1
Day31	10.5	30.7	6.83	4301	23154.4	1700	1000	0	37.1		55.4
Day32						0	1000	0	37.1		53.8
Day33	10.8	31.0	7.45	3734		0	1000	0	37.2		53.9
Day34						0	1000	0	36.7		53.6
Day35	10.4	30.6	7.72	3774		0	1000	0	37.2	74x40x50(L); 115x72(R)	52.9

Date, day after triggering dose of hCG; Hb, hemoglobin (g/dL); Hct, haematocrit; L, leucocytes x100/µL; albumin level, mg/dL; alb level, albumin level (mg/dL); HCG, human chorionic gonadotropin (mUI/mL); P, paracenteses (ml); T, thoracocenteses (ml); perf, perfusions (ml); albumin adm, albumin administration (albumin humaine C.R. 20%100ml); T, temperature (Celsius); patient weight (kg)

## DISCUSSION

OHSS is one of the most serious iatrogenic IVF complications to occur after ovarian stimulation. The pathogenesis of OHSS is not entirely understood but the most accepted mechanism is an overexpression of VEGF (Soares, 2012). This mediator of angiogenesis acts as a potent stimulator of vascular permeability and inflammation, which hypovolemia, hydroelectrolytic disorders, multi-organ failure and sometimes, death as potential consequences. High molecular weight plasma proteins could also accumulate in extravascular fluid under mediation of VEGF (Vandoorne *et al*., 2010; Abramov *et al*., 1999).

For severe cases of OHSS, multidisciplinary counsel and close clinical and biological monitoring are recommended (RCOG, 2016). The goal of treatment is to preserve intravascular blood volume. Guidelines and most studies (Sansone *et al*., 2011; RCOG, 2016; Shmorgun *et al*., 2011; Vandoorne *et al*., 2010) recommend use of macromolecules like albumin to maintain this intravascular fluid. Albumin is a blood-derived plasma expander, and it has been suggested that the binding and transport properties of human albumin result in binding and inactivation of the vasoactive intermediates responsible for the pathogenesis of OHSS. The osmotic function is the most well-known property of albumin, whose role it is to maintain intravascular volume in the event of capillary leakage, thus preventing the sequelae of hypovolemia, ascites and hemoconcentration. However, because vascular permeability is compromised, albumin could accumulate in the interstitium, drawing fluid into the extracellular space and leading to impaired re-expansion of the intravascular space (Shmorgun *et al.,* 2011; Kumar *et al*., 2013). Vandoorne *et al*. (2010) showed that large serum proteins, like albumin extravasate through large fenestration and vesiculo-vacuolar organelles and can accumulate selectively in the extravascular space in regions with elevated vascular permeability. In a pilot study in rabbits, Orvieto *et al*. (1998) showed that in animals treated with bovine serum albumin (BSA), body weight and ascites formation were higher than in animals not treated with BSA treatment. It appears that plasma albumin concentrations in patients with severe OHSS are significantly lower than in controls and ascitic fluid obtained from patients with OHSS contains large amounts of this protein (Abramov *et al*., 1999). Thus the potential protective action of albumin perfusion could be less than commonly believed, and it may even promote edema formation by further increase of extravascular colloid oncotic pressure (Kumar *et al*., 2013). Repeated ascitic fluid aspiration is usually necessary in case of severe OHSS in order to improve clinical parameters and to reduce hospitalization time (Qublan *et al*., 2012) and it is usually recommended to give human albumin in order to limit the rapid reconstitution of the third space. The quantity of aspirated fluid may vary up to significant value and consequently the quantity of albumin extracted. For this reason, albumin administration is recommended. The more albumin is administered the more will cross the capillary wall in this context of vascular hyperpermeability.

In our opinion, this theory could explain the vast quantities of liquid extracted by numerous paracenteses and thoracocenteses. Moreover, in our patient after stopping the albumin perfusion, the ascitis formation were ceased despite the 7^th^ week of pregnancy so with rise in hCG (hCG peaked between 56 and 68 day) (Braunstein *et al*., 1976). Albumin perfusion for OHSS also has other disadvantages, such as risks of exacerbation of ascites, nausea, vomiting, febrile reactions, allergic reactions, anaphylactic shock and possible virus and prion transmission (Ben-Chetrit *et al*., 2001).

Synthetic colloids such as gelatins, dextrans and hydroxyethyl starchs (HES) may also be utilized as plasma expanders. Conversely, in patients with increased vascular permeability, like human albumin, these colloid molecules may themselves leak into the interstitium and exert a reverse osmotic effect. HES is a macromolecule that has been extensively used in the treatment of severe OHSS, but very few studies have compared its efficacy with that of intravenous albumin. Many studies show the beneficial role of HES in maintaining plasma volume. However, Kissler et al. reported a case of detrimental role of HES in OHSS due to increase in capillary permeability with loss of this colloidal substance into the third space and prolong clinical OHSS symptoms (Kissler *et al*., 2001). Moreover, HES run a greater risk of adverse renal and coagulation effects than albumin and there is still uncertainly regarding their use in pregnancy.

Other treatment options for OHSS include oral antidiabetics (glibenclamide), dopamine and dopamine agonists in addition to crystalloids and colloids or anti-VEGF agents (Sansone *et al*., 2011; RCOG, 2016), but more studies are needed to assess the safety of these treatments if OHSS is associated with pregnancy.

## CONCLUSION

Albumin can be used for treatment of OHSS in case of pregnancy, but may result in persistence of the syndrome, as it could have been the case in our patient due to increase of vascular permeability including for macromolecules. Indeed, progressively increasing quantities of fluids were extracted despite use of supportive therapy until we stopped the albumin perfusion. We suggest that OHSS was iatrogenically maintained in the present case, as remission of the syndrome was observed after stopping albumin administration, even with increasing β-HCG. Treatment of OHSS is a delicate balance between invasive approaches, such as paracentesis, administration of colloids and minimal therapeutic intervention, particularly in case of pregnancy.

The detrimental role of albumin reported here in this paper could however be a coincidence too, since most cases of severe OHSS resolve after several weeks by themselves with or without application of colloids.
